# Use of oscillatory positive expiratory pressure (OPEP) devices to augment sputum clearance in COPD: An updated systematic review and meta-analysis

**DOI:** 10.1177/14799731261463730

**Published:** 2026-06-23

**Authors:** Ahmed A. Alzahrani, Saeed M Alghamdi, Mansour S. Majrshi, Ali M Alasmari, Surinder S Birring, Lizzie JF Grillo, Nicholas S Hopkinson

**Affiliations:** 1 National Heart and Lung Institute, 4615 Imperial College London, London, UK; 2 Faculty of Medical Rehabilitation Sciences, Taibah University, Madinah, Saudi Arabia; 3 Clinical Technology Department, Respiratory Care Program, Faculty of Applied Medical Sciences, 48058 Umm Al-Qura University, Makkah, Saudi Arabia; 4 Respiratory Care, University Hospital - King Abdulaziz University, Jeddah, Saudi Arabia; 5 Centre for Human & Applied Physiological Sciences, School of Basic & Medical Biosciences, Faculty of Life Sciences & Medicine, King’s College London, London, UK; 6 Physiotherapy Department, Royal Brompton Hospital, Guys and St Thomas’ NHS Foundation Trust, London, UK

**Keywords:** systematic review, COPD, oscillatory positive expiratory pressure, sputum clearance, lung function, meta-analysis

## Abstract

**Introduction:**

Effective airway clearance is crucial in COPD management, and oscillatory positive expiratory pressure (OPEP) devices are a potential adjunct therapy for this. However, their clinical efficacy remains uncertain due to limited trial data.

**Aim:**

To update our previous (2020) systematic review investigating the use of OPEP devices to augment sputum clearance in COPD.

**Methods:**

Randomised Clinical Trails s evaluating OPEP devices in COPD were identified from PubMed, CINAHL, Medline, Cochrane, and Embase (2020–2024). Outcomes included lung function, exercise capacity, exacerbations, and health-related quality of life (HRQoL), with pooled estimates calculated using random-effects models.

**Results:**

Twelve trials (741 participants) were included. OPEP devices significantly reduced exacerbations (Odds Ratio: 0.39) and improved exercise capacity (+49 m at 6MWD). Small improvements were observed in FVC%, while HRQoL changes were not statistically significant. Accumulating evidence suggests benefits for sputum clearance and reduced antibiotic use. Devices were generally well accepted and safe.

**Conclusion:**

OPEP devices appear to be safe and may reduce exacerbations, improve functional exercise capacity, and support sputum clearance in COPD.

## Introduction

Chronic obstructive pulmonary disease (COPD) is characterised by persistent airflow limitation and airway inflammation,^1^ with mucus hypersecretion commonly contributing to cough, dyspnoea, and impaired health-related quality of life^2^ Airway clearance may be supported by pharmacological therapies and non-pharmacological techniques, including oscillatory positive expiratory pressure (OPEP) devices, which generate expiratory oscillations and positive pressure to mobilise secretions and reduce airway collapse.^3^ The positive expiratory pressure produced also helps prevent airway collapse, enhance collateral ventilation, and support secretion clearance. Adjustable valves in OPEP devices allow modification of expiratory resistance. A previous systematic review reported potential short-term benefits of OPEP therapy on sputum clearance and exercise capacity; however, conclusions were limited by small sample sizes, short intervention durations, methodological heterogeneity, and limited evidence for clinically important outcomes such as exacerbations.^4^ Since that review, several additional randomised controlled trials have been published, including studies with larger sample sizes, longer intervention durations, and broader assessment of clinically relevant outcomes, substantially expanding the available evidence base. These newer studies provide an opportunity to address limitations identified in the earlier review and determine whether emerging evidence alters previous conclusions regarding the effectiveness of OPEP devices in COPD. An updated systematic review is therefore warranted to synthesise this expanded evidence base and reassess the role of OPEP therapy in COPD management.^5^

## Methods

This update to our 2020 systematic review^6^ was registered on PROSPERO (CRD 42024554789). The review was conducted in accordance with the PRISMA Statement guidelines (Table 1).^7^

**Table 1.** PICO (Population, Intervention, Comparator, Outcomes) framework for studies assessing the effectiveness of OPEP/PEP devices in adults with COPD.

Population	Adults with COPD
Intervention	OPEP/PEP devices
Comparator	Usual care, sham, no intervention, or alternative airway clearance techniques
Outcomes	HRQoL, symptoms and AECOPD
	Number of exacerbations
	Antibiotic use
	Exercise capacity
	Lung function
	Sputum clearance

### Inclusion criteria

Eligible studies were randomised controlled or crossover trials involving adults with COPD as the primary respiratory diagnosis, defined according to established diagnostic criteria (post-bronchodilator FEV_1_/FVC <0.70 consistent with Global Initiative for Chronic Obstructive Lung Disease recommendations, evaluating OPEP devices. Mixed respiratory populations were excluded unless COPD-specific data were reported separately.^4,5^

### Exclusion criteria

Studies were excluded if they were not in English. Studies involving mixed respiratory populations were excluded unless outcome data for participants with COPD could be extracted separately. Studies that included participants with COPD but did not report outcomes specific to COPD were excluded. Additionally, studies that assessed only a single treatment session were excluded, as this review focused on interventions involving repeated use and clinically meaningful outcomes that require sustained treatment exposure, rather than short-term physiological effects observed after a single session.

### Search strategy

Searches were conducted across PubMed, CINAHL, Medline (Ovid), Cochrane, and Embase databases. In consultation with a librarian, additional manual searches were performed on the reference lists of relevant primary studies and reviews to identify any additional studies that may not have been captured in the original search (Appendix 1).

### Search procedures

The main author conducted the search, after which all articles were imported into EndNote, and duplicates were removed using Rayyan QCRI. Titles and abstracts of all articles were screened. A manual search of reference lists from relevant studies was performed to identify potentially suitable articles that might have been missed from the database search. Subsequently, a full-text review of all included articles was conducted. Any study that did not meet the inclusion criteria was excluded, and reasons for exclusion were recorded according to the PRISMA flowchart (Appendix 2).

### Data extraction

Data related to studies, populations, and outcomes were extracted independently by two reviewers using a standardised data-extraction form developed for this review. Study characteristics included authors’ names, year of publication, country, study duration, and research design. Population characteristics included the number of subjects, age, gender, BMI, GOLD grade if reported, Forced Expiratory Volume in one second as a percentage of predicted (FEV1%), number of COPD patients who consented to participate; number of index-year exacerbations (mean ± SD), sputum clearance, health-related quality of life (HRQoL), physical activity, and fatigue. After extraction, the two reviewers cross-checked all entries for accuracy and completeness. Any discrepancies were resolved through discussion and, when necessary, consultation with a third reviewer. This process ensured consistency and minimised errors in the final dataset.

### Data analysis

The synthesis of results centred on evaluating primary outcomes, including health-related quality of life (HRQoL), COPD symptoms, frequency of acute exacerbations, lung function parameters, and exercise capacity. Additionally, patient adherence indicators such as acceptance, completion, and dropout rates were assessed. A meta-analysis was conducted to calculate pooled differences and 95% confidence intervals (CIs) for key outcomes, comparing the OPEP group with the control group. A random-effects model was applied to ensure conservative estimates. Continuous outcomes were presented as mean differences (D), and standardised mean differences (SMD) were used when outcomes were measured on varying scales. For categorical data, odds ratios (OR) were employed. Study heterogeneity was examined using the I^2^ statistic, while publication bias was evaluated through funnel plots of included studies, and certainty of evidence for key outcomes was additionally assessed using the GRADE (Grading of Recommendations Assessment, Development and Evaluation) approach (Appendices 3, 4). Analyses were conducted with the Cochrane Collaboration’s Review Manager Software RevMan (5.4.1). The acceptance rate was calculated as the proportion of participants who consented to participate out of all those approached for the trial. The completion rate was defined as the number of participants who completed the study relative to those enrolled, and the dropout rate was calculated based on the number who withdrew from each treatment arm as a fraction of those who initially consented.^8^ An additional meta-analysis was undertaken to estimate pooled differences and 95% CIs for acceptance, completion, and dropout rates between the OPEP and control groups. Rates were weighted by study sample size and combined using random-effects models. All rates are reported as proportions with 95% CIs.

## Results

**Figure 1.**
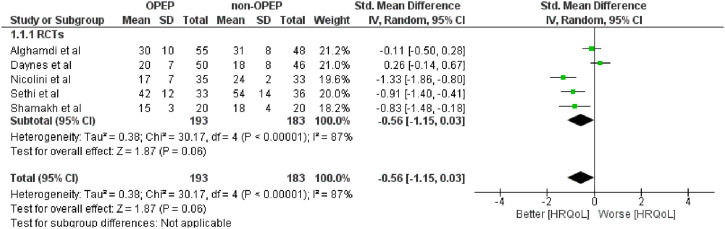
Forest plot comparing HRQoL measures (CAT and SGRQ) scores in OPEP interventions vs non-OPEP interventions.

Between 2020 and 2024, a systematic search of PubMed and Embase identified 91 records, of which 11 duplicates were removed. After screening 80 titles and abstracts, 69 records were excluded, leaving 11 articles for full-text review. Four additional randomised controlled trials met the eligibility criteria and were included alongside the eight studies from the previous review^6^ (Figure 1) (Table 2).

**Table 2.** New and old studies include.

Author and year	Patient group design	OPEP device	Treatment duration	Follow-up	Control groups	Results of OPEP group compared to corresponding groups
Shamakh et al., 2020	Moderate COPD, age 40-65, BMI 18-24.9 Randomised Controlled Trial	Acapella, Threshold PEP	6 months	Acute exacerbation and 6 months	Active Cycle of Breathing Technique (ACBT) only	FEV1 and FEV1/FVC improved; Acapella and PEP were more effective than ACBT alone. ΔFEV1: +10.49% vs +2.64% (p<0.001); ΔFEV1/FVC: +8.52% vs +3.71% (p=0.001); Δ6MWD: +20.09% vs +3% (p=0.004).
						Significant improvement in lung function and exercise capacity.
Xu et al., 2023	Pre-COPD to severe COPD, age 18-80 Randomised Controlled Trial	PEP device (5 cmH2O)	2 months, 4 hours/day	2 months	Sham PEP device	Improved 6MWD, end-dyspnea, end-fatigue, no adverse events.
						Δ6MWD: +71.67m (p<0.001); End- dyspnoea (p=0.002); End-fatigue (p=0.022).
						Safe, well-tolerated, and improved functional capacity.
Alghamdi et al., 2023	COPD with daily sputum production,FEV_1_ 1.08 L (38% predicted); FVC 3.0 L (86% predicted); RV 211% predicted.Randomised Controlled Trial	Acapella	3 months	3 months	Usual care (ACBT)	Improved LCQ score, cough frequency, fatigue, quality of life. ΔLCQ: +1.03 (p=0.03); ΔFACIT: +4.68 (p<0.001); ΔEQ-5D: +4.00 (p=0.04); Reduced cough frequency (p<0.001); Exacerbations: 32% vs 54% (p=0.029).
						Significant improvement in symptoms and quality of life.
Daynes et al., 2022	Symptomatic COPD, FEV1 48% predicted Double-blinded, Sham-controlled Trial	(Aerosure)	8 weeks, 3x/day	8 weeks	Sham device	Improved inspiratory pressure, no significant difference in dyspnoea scores. ΔPImax: +5.26 (p=0.05); Improvement in inspiratory pressure, OPEP therapy significantly improved inspiratory muscle strength p=0.05, but this did not translate into significant improvements in dyspnoea p=0.24
[studies from previous review]						
Aggarwal (2010)	Hospitalised AECOPD RCT	Flutter n=15	15-mins, 3x per day, for 5 days	Every day	Control 1: ACBT n=15	Flutter and ACBT had the same effect on lung function compared to pursed lip breathing (ΔPEFR; +30 L/min)
					Control 2: pursed lip breathing n=15	Flutter reduced hospital stay compared to ACBT and pursed lip breathing (3/5/5 days)
Cegla (2002)	Stable COPD FEV1 40±14% RCT	RC-Cornet plus UC n=25	>5-mins, 3x per day, for 2 years	Every 3 months	UC n=25	RC-Cornet had the same effect as UC on lung function (ΔFVC%; predicted +2%)
						RC-Cornet reduced antibiotic use compared to UC (12/25 vs 24/25)
						RC-Cornet reduced exacerbations over 2 years compared to UC (5/25 vs 12/25)
						RC-Cornet had the same effect as UC on hospital stays (17 vs 18 days).
McCarroll (2005)	Stable COPD with hypersecretion RCT	Acapella plus PR n=12	10 - mins, 2x per week, for 8 weeks	Every 4 weeks	Control 1: UC n=11	Acapella had the same effect as UC & PR on lung function (Δ FEV1 and PEFR; +0.28 L/min and +16 L/min)
					Control 2: PR n=12 (2 sessions per week, for 8 weeks)	Improvement in exercise capacity did not differ significantly between UC and PR (Δ6MWD; +44 m vs +54 m)
Nicolini (2018)	Stable COPD FEV1=31±10% RCT	Lung Flute plus UC n=40	30-mins, 2x per day, for 12 days and then 26 weeks follow up	Every 4 weeks	Control 1: Flutter n=40 (30-mins, 2x per day, for 12 days and then 26 weeks follow up)	Lung Flute and Flutter reduced exacerbations compared to UC (7/40 vs 9/40 vs 11/40)
						Lung Flute and Flutter improved exercise capacity vs UC (Δ6MWD; +18.4 m/+11.5 m/-4.8 m)
						Lung Flute, Flutter, and UC; no difference in cough or sputum clearance (Δ BCSS score; -3/-3.1/-3.5)
					Control 2: UC n=40	Lung Flute and Flutter improved HRQoL compared to UC (Δ CAT score; -7.5/-6.4/-1.6)
						Lung Flute and Flutter reduced dyspnoea compared to UC (ΔMMRC score; -0.6/-0.4/+0.1)
Sethi (2015)	Stable COPD with sputum production, FEV1 50±3% RCT	Lung Flute plus UC n=33	5-mins, 2x per day for 26 weeks	Every 8 weeks	UC n=36	Lung Flute reduced symptoms compared to UC (Δ CCQ score; -0.23 vs +0.01)
						Lung Flute improved HRQoL compared to UC (ΔSGRQ score; -3.23 vs -1.85, p=0.03)
						Lung Flute reduced exacerbations compared to UC (6/33 vs 14/36, p=0.03)
						Lung Flute improved exercise capacity compared to UC (Δ6MWD; +7 m vs -42 m)
Svenningsen (2016)	Stable COPD- sputum producer vs non-sputum producer FEV1 60±18% RXT	Aerobika plus UC n=27	20-mins, 4x per day, for 3 weeks (one-week intervention, one-week washout, and one-week UC)	Not reported	UC	Aerobika improved lung function compared to UC (Δ FVC% predicted; +6%, p=0.005)
						Aerobika improved HRQoL compared to UC (ΔSGRQ score; -9, p=0.01).
						Aerobika improved sputum clearance compared to UC (ΔPEQ-ease-bringing-up-sputum; -1.2, p = 0.005)
						Aerobika improved exercise capacity compared to UC (Δ 6MWD; +19 m, p = 0.04)
						Aerobika improved regional ventilation compared to UC (Δ 3He MRI ventilation deficit percent; -1%).
Weiner, (1996)	Stable COPD FEV1 35±8.5% predicted. RCT	Flutter n=10	10 mins, 4-8x per day for 3 months.	Not reported	Sham Flutter 10 mins, 4-8 times/day for 3 months. n=10	Flutter and Sham Flutter no effect on lung function (ΔFVC% predicted +2% vs +2%)
						Flutter improved exercise capacity vs Sham Flutter (Δ12-minute walk distance; +649 m vs +538 m)
Wolkove, (2004)	Stable COPD with sputum production and smoking history FEV1 50±15% RXT	Flutter plus UC n=15	10-mins, 4x per day, for 1 week	Every week	Sham Flutter 10-mins, 4x per day, for 1 week	Flutter improved lung function vs Sham Flutter (Δ FVC%; +24%, p=0.05)
						Flutter improved exercise capacity vs Sham Flutter (Δ 6MWD; +10 m, p=0.05)
						Flutter reduced dyspnoea vs Sham Flutter (Δ Borg scale; +1, p=0.05)

### Characteristics of included studies

Twelve randomised controlled trials involving 741 participants were included in this updated review, comprising eight studies from the previous review and four newly identified trials. Sample sizes ranged from 25 to 122 participants, with most studies enrolling patients with stable COPD, although a small number included participants during acute exacerbations. Participants represented a range of disease severity and symptom burden, with some studies specifically recruiting individuals with chronic sputum production. A variety of OPEP and PEP devices were evaluated, including Acapella, Flutter, Aerobika, Lung Flute and RC-Cornet, with intervention durations ranging from several weeks to two years. Comparator groups included usual care, sham devices, and alternative airway clearance or physiotherapy interventions. Overall, studies were heterogeneous in participant characteristics, intervention protocols and follow-up duration. Detailed study and participant characteristics are summarised in (Table 2).

### Risk of bias assessment

Risk of bias was assessed using the Cochrane Risk of Bias assessment tool^9^ across all 12 included studies. Most studies adequately described their random sequence generation and allocation concealment, indicating a generally low risk of selection bias. However, blinding of participants and personnel was rarely achievable because the interventions involved physical devices, leading to a consistently high risk of performance bias. Several studies also demonstrated unclear or high risk regarding the blinding of outcome assessment, which may introduce detection bias. Variability was observed in incomplete outcome data, with some studies lacking transparent reporting of attrition or missing data management. Additionally, selective reporting was noted in a number of studies where prespecified outcomes were not fully reported. Overall, the combined assessment of all 12 studies highlights a generally low risk of bias in selection-related domains but persistent limitations in blinding and reporting quality (Appendix 3) (Table 3).

**Table 3.** Risk of bias assessment of included studies.

Study	Random sequence generation	Allocation concealment	Blinding participants/personnel	Blinding outcome assessment	Incomplete outcome data	Selective reporting	Other bias
Aggarwal et al.	Unclear	Unclear	High	High	High	High	High
Alghamdi et al.	Low	Low	Low	Low	Low	Low	Unclear
Cegla et al.	Unclear	Unclear	High	High	Low	High	High
Daynes et al.	Low	Low	Unclear	Low	Low	Low	Low
McCarroll et al.	Low	Low	Unclear	Low	Unclear	Low	Unclear
Nicolini et al.	Low	Low	Low	Low	Unclear	Low	Unclear
Sethi et al.	Low	Low	High	High	Low	Low	High
Shamakh et al.	Low	Low	High	Low	Low	Low	Low
Svenningsen et al.	Low	Low	High	Low	Unclear	Low	Unclear
Weiner et al.	Low	Unclear	Unclear	Low	Unclear	High	Unclear
Wolkove et al.	Unclear	Low	High	High	Unclear	Low	Unclear
Xu et al.	Low	Low	High	Low	Low	Low	Unclear

### Acceptance, completion, and dropout rates

Across the twelve included randomised controlled trials evaluating PEP and OPEP devices in COPD, acceptance, completion, and dropout rates were generally favourable. Overall, 350 participants completed their assigned intervention, while 31 (approximately 9%) withdrew before study completion. The primary reasons for dropout included loss to follow-up (66%), COPD exacerbations (16%), death (6%), back pain (6%), MRI-related discomfort (3%), and unknown reasons (3%). From these studies, the unweighted acceptance, completion, and dropout rates were 82%, 91%, and 6%, respectively.

### Stable COPD

#### HRQoL, symptoms and AECOPD

Health-related quality of life (HRQoL) was reported in five randomised controlled trials.^10–14^ When pooled, the meta-analysis for improved HRQoL was not statistically significant in patients using OPEP devices compared with non-OPEP controls (SMD = –0.56; 95% CI: –1.15 to 0.03; p = 0.06) although the direction of effect favoured the OPEP intervention. Substantial heterogeneity was observed (I^2^ = 87%), suggesting considerable variability in effect estimates, likely arising from differences in study populations, intervention duration, device characteristics, and the HRQoL measurement tools used (Figure 1).

#### Number of exacerbations

**Figure 2.**
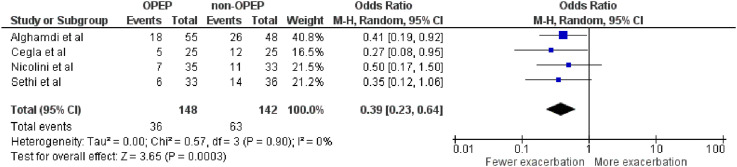
Forest plot comparing exacerbation events of OPEP use vs usual care in stable COPD.

Exacerbations were reported in four randomised controlled trials.^10,12,13,15^ The pooled analysis showed that OPEP device use was associated with a significant reduction in exacerbation events compared with non-OPEP controls (OR = 0.39; 95% CI: 0.23 to 0.64; p = 0.0003) the pooled analysis showed that OPEP device use was associated with a significant reduction in exacerbation events compared with non-OPEP controls (OR = 0.39; 95% CI: 0.23 to 0.64; p = 0.0003). This corresponds to an estimated 61% reduction in exacerbation risk among patients using OPEP devices. There was no observed heterogeneity (I^2^ = 0%), indicating a consistent effect across studies despite differences in sample size, device type, and patient characteristics. As such, there is moderate confidence, according to the GRADE assessment, that OPEP devices reduce exacerbation frequency, and the true effect is likely to be close to the observed estimate (Figure 2).

#### Antibiotic use

Antibiotic use was inconsistently reported across the included studies. Only one long-term trial^16^ evaluated antibiotic consumption as a formal outcome and found that twice-daily use of the RC-Cornet device over a 2-year period significantly reduced the number of patients requiring at least one course of antibiotics compared with the control group (13/25 vs. 24/25; OR = 0.05; 95% CI: 0.01 to 0.38; p = 0.005). None of the remaining eleven studies, including the four newly added trials,^10,11,14,17^ reported antibiotic use as an outcome measure. Consequently, while the available evidence suggests a potential reduction in antibiotic use with long-term OPEP therapy, the certainty of this finding is limited due to the reliance on a single study and the absence of supporting data from other trials.

#### Exercise capacity

**Figure 3.**
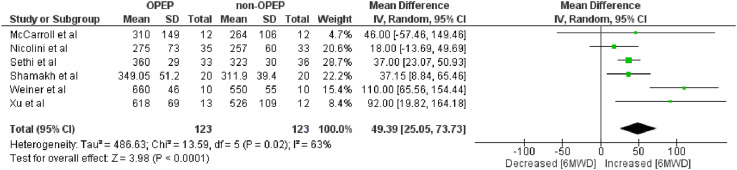
Forest plot comparing exercise capacity measured with 6MWD (in metres) in OPEP interventions vs non-OPEP interventions.

Six randomised controlled trials^12–14,17–19^ reported changes in 6-minute walk distance (6MWD). The pooled analysis demonstrated a significant improvement in exercise capacity among patients using OPEP devices compared with non-OPEP controls (MD = 49.39 m; 95% CI: 25.05 to 73.73 m; p < 0.0001). Both newly added studies^14^ and^17^ contributed to the positive direction of effect, with^17^ reporting one of the largest individual improvements (Figure 3*).*

#### Lung function

**Figure 4.**
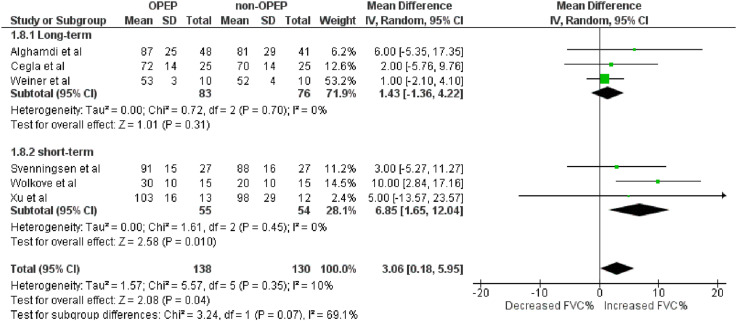
Forest plot comparing PFT measured with FVC% long-short term in OPEP interventions vs non-OPEP interventions.

Forced vital capacity percentage (FVC%) was reported in six RCTs.^10,15–17,19,20^ The pooled analysis demonstrated a statistically significant improvement in FVC% among patients using OPEP devices compared with non-OPEP controls (MD = 3.06%; 95% CI: 0.18 to 5.95; p = 0.04). Subgroup analysis showed that long-term studies did not demonstrate significant improvement (MD = 1.43%; 95% CI: –1.36 to 4.22), whereas short-term studies produced a clinically meaningful increase in FVC% (MD = 6.85%; 95% CI: 1.65 to 12.04). The newly added trials^10^ in the long-term group and^17^ in the short-term group contributed to the overall pooled effect in their respective subgroups. Heterogeneity was low overall (I^2^ = 10%) but moderate within the short-term subgroup (I^2^ = 69%), likely reflecting differences in intervention duration, baseline lung function, and device characteristics (Figure 4).

#### Sputum clearance

Sputum clearance was evaluated in only two of the twelve included studies,^10,20,21^ each using different oscillatory PEP devices and outcome measures. An earlier RCT^20^ assessed sputum clearance over a 3-week period using the Aerobika device and reported a significantly greater improvement compared with usual care, as reflected by lower PEQ ease-of-bringing-up-sputum scores (mean ± SD: 2.70 ± 1.10 vs. 3.60 ± 0.50; p = 0.003), where a lower score indicates better clearance. In contrast, a more recent trial^10^ using the Acapella device demonstrated a statistically and clinically significant improvement in sputum clearance, showing a mean increase of 0.50 on the sputum frequency scale (95% CI: 0.27–0.60; p < 0.001), compared with a negligible change in the control group (+0.06). Importantly, none of the included studies reported objective sputum clearance outcomes such as sputum volume or weight, and assessment was limited to patient-reported or symptom-based measures.

## Discussion

In COPD, improving sputum clearance remains a key therapeutic objective, given its association with daily symptom burden, health-related quality of life, and risk of acute exacerbations.^22^ Findings from this updated review suggest that OPEP devices may improve several clinically relevant outcomes, including symptoms, exacerbation frequency, exercise capacity and sputum clearance. A reduction in antibiotic use was also observed in the only long-term trial reporting this outcome.^20^

This update included four additional randomised controlled trials, increasing the evidence base from eight to twelve studies and expanding the pooled sample from 339 to 741 participants. While a previous 2020 review^6^ suggested potential benefits of OPEP devices for reducing exacerbations and improving exercise capacity, those findings were based on a limited number of predominantly short-term studies with low-certainty evidence. The present update strengthens and extends those earlier observations through a broader evidence base, more precise pooled effect estimates, and more consistent signals of benefit.

Exacerbation outcomes were generally modest but directionally consistent, with pooled results demonstrating a reduction in exacerbation risk among patients using OPEP devices. However, the number of contributing studies remains small, most interventions were relatively short in duration, and methodological limitations, particularly related to blinding and outcome reporting, temper confidence in these findings. Health-related quality of life showed a non-significant trend favouring OPEP therapy, though with substantial heterogeneity likely reflecting variation in patient populations, intervention duration, device type and outcome measures used.^23^ This variability highlights the need for greater standardisation of outcome assessment in future trials.

Exercise capacity improved across studies reporting 6-minute walk distance,^12,17,18,24–26^ with pooled gains of approximately 49 m, exceeding commonly reported minimal clinically important difference thresholds for COPD (approximately 25–35 m), supporting the clinical relevance of this finding.^23^ Longer-duration interventions appeared to show particularly consistent benefits, suggesting that sustained treatment exposure may be important.

Although sputum production is a prominent and distressing symptom for many individuals with COPD, it remains relatively neglected in current COPD guidelines.^4,22,27,28^ Neither the Global Initiative for Chronic Obstructive Lung Disease (GOLD) nor the American Thoracic Society/European Respiratory Society (ATS/ERS) guidelines make explicit recommendations on sputum clearance techniques,^21,22,29^ whereas the National Institute for Health and Care Excellence (NICE) guidelines briefly suggest the use of PEP devices or active cycle of breathing techniques for “excessive” sputum,^30^ without defining the threshold at which these should be implemented. The findings of this review suggest that patients with chronic sputum production may derive the greatest benefit from OPEP therapy. Only one included study differentiated sputum producers from non-producers, and those with regular sputum demonstrated more pronounced improvements.^20^ Future research should therefore adopt clearer phenotyping to identify the subgroups most likely to respond.

A range of OPEP and PEP devices were evaluated, with generally high acceptance and completion rates, suggesting good patient acceptability. The largest improvements in symptoms, HRQoL, and exacerbations were seen with Acapella, Lung Flute, and Aerobika, whereas fewer benefits were observed with Flutter ^10 13^. This may reflect differences in device mechanisms, including adjustable resistance, oscillation frequency, and ease of use.^31^

Dropouts in OPEP trials were commonly related to COPD exacerbations, highlighting the need for flexible study designs that can accommodate temporary interruptions or modification of device use. In addition, factors such as cognitive ability, physical technique, and the level of training provided to patients may influence adherence and overall treatment success. Real-world application of OPEP devices exhibits considerable variability, and healthcare professionals themselves express uncertainty concerning optimal thresholds for device prescription and patient selection. This uncertainty reflects challenges similar to those identified in the trials included in this review.^32^

Overall, this review highlights gaps in the existing evidence base and identifies priorities for future research. These include stratifying patients by sputum production, standardising outcome measures, evaluating longer-term effectiveness, and conducting direct comparisons between device types.

### Limitations

This review has several limitations. Many trials were at high risk of performance and detection bias due to limited blinding in device-based interventions and incomplete reporting of missing data and adherence, which reduced confidence in the findings. Outcome measures varied widely, contributing to heterogeneity and limiting comparability. Most studies were small and short-term, restricting assessment of long-term effectiveness. Few trials stratified patients by sputum burden, and no direct comparisons between OPEP devices were available.

## Conclusion

This updated review suggests that OPEP devices may reduce exacerbations, improve exercise capacity, enhance sputum clearance, and produce short-term benefits for lung function in COPD, particularly in patients with chronic sputum production.

## Supplemental material

Supplemental material - Use of oscillatory positive expiratory pressure (OPEP) devices to augment sputum clearance in COPD: An updated systematic review and meta-analysis Supplemental material for Use of oscillatory positive expiratory pressure (OPEP) devices to augment sputum clearance in COPD: An updated systematic review and meta-analysis by Ahmed A. Alzahrani, Saeed M Alghamdi, Mansour S. Majrshi, Ali M Alasmari, Surinder S Birring, Lizzie Grillo, Nicholas S Hopkinson in Chronic Respiratory Disease.

Supplemental material - Use of oscillatory positive expiratory pressure (OPEP) devices to augment sputum clearance in COPD: An updated systematic review and meta-analysis Supplemental material for Use of oscillatory positive expiratory pressure (OPEP) devices to augment sputum clearance in COPD: An updated systematic review and meta-analysis by Ahmed A. Alzahrani, Saeed M Alghamdi, Mansour S. Majrshi, Ali M Alasmari, Surinder S Birring, Lizzie Grillo, Nicholas S Hopkinson in Chronic Respiratory Disease.

Supplemental material - Use of oscillatory positive expiratory pressure (OPEP) devices to augment sputum clearance in COPD: An updated systematic review and meta-analysis Supplemental material for Use of oscillatory positive expiratory pressure (OPEP) devices to augment sputum clearance in COPD: An updated systematic review and meta-analysis by Ahmed A. Alzahrani, Saeed M Alghamdi, Mansour S. Majrshi, Ali M Alasmari, Surinder S Birring, Lizzie Grillo, Nicholas S Hopkinson in Chronic Respiratory Disease.

Supplemental material - Use of oscillatory positive expiratory pressure (OPEP) devices to augment sputum clearance in COPD: An updated systematic review and meta-analysis Supplemental material for Use of oscillatory positive expiratory pressure (OPEP) devices to augment sputum clearance in COPD: An updated systematic review and meta-analysis by Ahmed A. Alzahrani, Saeed M Alghamdi, Mansour S. Majrshi, Ali M Alasmari, Surinder S Birring, Lizzie Grillo, Nicholas S Hopkinson in Chronic Respiratory Disease.

Supplemental material - Use of oscillatory positive expiratory pressure (OPEP) devices to augment sputum clearance in COPD: An updated systematic review and meta-analysis Supplemental material for Use of oscillatory positive expiratory pressure (OPEP) devices to augment sputum clearance in COPD: An updated systematic review and meta-analysis by Ahmed A. Alzahrani, Saeed M Alghamdi, Mansour S. Majrshi, Ali M Alasmari, Surinder S Birring, Lizzie Grillo, Nicholas S Hopkinson in Chronic Respiratory Disease.

Supplemental material - Use of oscillatory positive expiratory pressure (OPEP) devices to augment sputum clearance in COPD: An updated systematic review and meta-analysis Supplemental material for Use of oscillatory positive expiratory pressure (OPEP) devices to augment sputum clearance in COPD: An updated systematic review and meta-analysis by Ahmed A. Alzahrani, Saeed M Alghamdi, Mansour S. Majrshi, Ali M Alasmari, Surinder S Birring, Lizzie Grillo, Nicholas S Hopkinson in Chronic Respiratory Disease.
