# Supplementary material for: Use of oscillatory positive expiratory pressure (OPEP) devices to augment sputum clearance in COPD: An updated systematic review and meta-analysis
Source: Chron Respir Dis. 2026 Jun 23;23:14799731261463730. doi: 10.1177/14799731261463730 (PMC13305770; doi:10.1177/14799731261463730)
Supplement: Supplemental material - Use of oscillatory positive expiratory pressure (OPEP) devices to augment sputum clearance in COPD: An updated systematic review and meta-analysis [file sj-pdf-1-crd-10.1177_14799731261463730.pdf]

Appendix 1 Search strategies of specific database

Ovid MEDLINE (2020 to March 2024)

1. Pulmonary Disease, Chronic Obstructive/ or lung diseases, obstructive/ or Obstructive
airway disease/ or Chronic obstructive lung disease/
2. Emphysema/ or Pulmonary Emphysema/ or Bronchitis, Chronic/ or Bronchitis/
3. Chronic Obstructive Pulmonary* Disease*.mp.
4. Chronic Obstructive lung* disease*.mp.
5. (COPD or COAD or chronic bronchi* or emphysema* or hyperlucent lung*).mp.
6. or/1-5
7. airway* clearance device*.mp.
8. airway* clearance therapy.mp.
9. sputum* clearance technique*.mp.
10. chest clearance*.mp.
11. Acapella*.mp.
12. Aerobika*.mp.
13. lung flute*.mp.
14. positive* expiratory pressure*.mp.
15. positive expiratory pressure therapy*.mp.
16. Oscillatory Positive Expiratory Pressure*.mp.
17. OPEP*.mp.
18. or/7-17
19. 6 and 18
20. limit 19 to (english language and humans)

EMBASE (2020 to March 2024)

1. Pulmonary Disease, Chronic Obstructive/ or lung diseases, obstructive/ or
Obstructive airway disease/ or Chronic obstructive lung disease/
2. Emphysema/ or Pulmonary Emphysema/ or Bronchitis, Chronic/ or Bronchitis/
3. Chronic Obstructive Pulmonary* Disease*.mp.
4. Chronic Obstructive lung* disease*.mp.
5. (COPD or COAD or chronic bronchi* or emphysema* or hyperlucent lung*).mp.
6. or/1-5
7. airway* clearance device*.mp.
8. airway* clearance therapy.mp.
9. sputum* clearance technique*.mp.
10. chest clearance*.mp.
11. Acapella*.mp.
12. Aerobika*.mp.
13. lung flute*.mp.

14.	positive* expiratory pressure*.mp.
15.	positive expiratory pressure therapy*.mp.
16.	Oscillatory Positive Expiratory Pressure*.mp.
17.	OPEP*.mp.
18.	or/7-17
19.	6 and 18
20.	limit 19 to (english language and humans)

CINAHL (EBSCO) (2020 to March 2024)

1.	(MM “Lung Diseases, Obstructive”) OR (MM “Pulmonary Disease, Chronic Obstructive”)
2.	(MM “Emphysema”) OR (MM “Bronchitis, Chronic”) OR (MM “Bronchitis”)
3.	S1 OR S2
4.	(TX “OPEP*”) OR (MM” Oscillatory Positive Expiratory Pressure”)
5.	(MM” positive expiratory pressure therapy”) OR (MM” positive expiratory pressure”)
6.	(TX” lung flute”) OR (MM” lung flute”)
7.	(TX” Flutter device”) OR (MM” Flutter device”)
8.	(MM” Aerobika”) OR (MM” Acapella”)
9.	(TX” chest clearance technique*”) OR (TX” sputum clearance technique*”)
10.	(TX” airway clearance therapy”) OR (MM” airway clearance device”)
11.	S4 OR S5 OR S6 OR S7 OR S8 OR S9 OR S10
12.	S3 AND S11
13.	Limit S12 to Language: English

Cochrane Database of Systematic Reviews – 2020 to March 2024

#1 (airway clearance device):ti,ab,kw (Word variations have been searched)
#2 airway clearance technique
#3 airway clearance therapy
#4 chest clearance technique
#5 sputum clearance technique
#6 Acapella
#7 Aerobika
#8 Flutter device
#9 lung flute
#10 positive expiratory pressure
#11 positive expiratory pressure therapy
#12 Oscillatory Positive Expiratory Pressure
#13 OPEP
#14 #1 or #2 or #3 or #4 or #5 or #6 or #7 or #8 or #9 or #10 or #11 or #12 or #13
#15 Chronic Obstructive Pulmonary Disease

172
#16 Chronic Obstructive lung disease
#17 COPD
#18 #15 or #16 or #17
#19 #14 and #18

PubMed – 2020 to 2024

**1.** airway [All Fields] AND clearance [All Fields] AND ("equipment and supplies"[MeSH
Terms] OR ("equipment"[All Fields] AND "supplies"[All Fields]) OR "equipment and
supplies"[All Fields] OR "device"[All Fields])
**2.** airway [All Fields] AND clearance [All Fields] AND technique [All Fields]
**3.** airway [All Fields] AND clearance [All Fields] AND ("therapy"[Subheading] OR
"therapy"[All Fields] OR "therapeutics"[MeSH Terms] OR "therapeutics"[All Fields])
**4.** ("thorax"[MeSH Terms] OR "thorax"[All Fields] OR "chest"[All Fields]) AND
clearance [All Fields] AND technique[All Fields]
**5.** ("sputum"[MeSH Terms] OR "sputum"[All Fields]) AND clearance [All Fields] AND
technique [All Fields]
**6.** Acapella[All Fields]
**7.** Aerobika[All Fields]
**8.** Flutter[All Fields] AND ("equipment and supplies"[MeSH Terms] OR ("equipment"[All
Fields] AND "supplies"[All Fields]) OR "equipment and supplies"[All Fields] OR
"device"[All Fields])
**9.** ("lung"[MeSH Terms] OR "lung"[All Fields]) AND flute[All Fields]
**10.** positive[All Fields] AND ("exhalation"[MeSH Terms] OR "exhalation"[All Fields] OR
"expiratory"[All Fields]) AND ("pressure"[MeSH Terms] OR "pressure"[All Fields])
**11.** positive[All Fields] AND ("exhalation"[MeSH Terms] OR "exhalation"[All Fields] OR
"expiratory"[All Fields]) AND ("pressure"[MeSH Terms] OR "pressure"[All Fields])
AND ("therapy"[Subheading] OR "therapy"[All Fields] OR "therapeutics"[MeSH
Terms] OR "therapeutics"[All Fields])
**12.** Oscillatory[All Fields] AND Positive[All Fields] AND ("exhalation"[MeSH Terms] OR
"exhalation"[All Fields] OR "expiratory"[All Fields]) AND ("pressure"[MeSH Terms] OR
"pressure"[All Fields])
**13.** OPEP[All Fields]
**14.** "pulmonary disease, chronic obstructive"[MeSH Terms] OR ("pulmonary"[All Fields]
AND "disease"[All Fields] AND "chronic"[All Fields] AND "obstructive"[All Fields]) OR
"chronic obstructive pulmonary disease"[All Fields] OR ("chronic"[All Fields] AND
"obstructive"[All Fields] AND "pulmonary"[All Fields] AND "disease"[All Fields])
**15.** "pulmonary disease, chronic obstructive"[MeSH Terms] OR ("pulmonary"[All Fields]
AND "disease"[All Fields] AND "chronic"[All Fields] AND "obstructive"[All Fields]) OR
"chronic obstructive pulmonary disease"[All Fields] OR ("chronic"[All Fields] AND
"obstructive"[All Fields] AND "lung"[All Fields] AND "disease"[All Fields]) OR "chronic
obstructive lung disease"[All Fields]
