# Supplementary material for: Use of oscillatory positive expiratory pressure (OPEP) devices to augment sputum clearance in COPD: An updated systematic review and meta-analysis
Source: Chron Respir Dis. 2026 Jun 23;23:14799731261463730. doi: 10.1177/14799731261463730 (PMC13305770; doi:10.1177/14799731261463730)
Supplement: Supplemental material - Use of oscillatory positive expiratory pressure (OPEP) devices to augment sputum clearance in COPD: An updated systematic review and meta-analysis [file sj-pdf-2-crd-10.1177_14799731261463730.pdf]

Appendix2 PRISMA

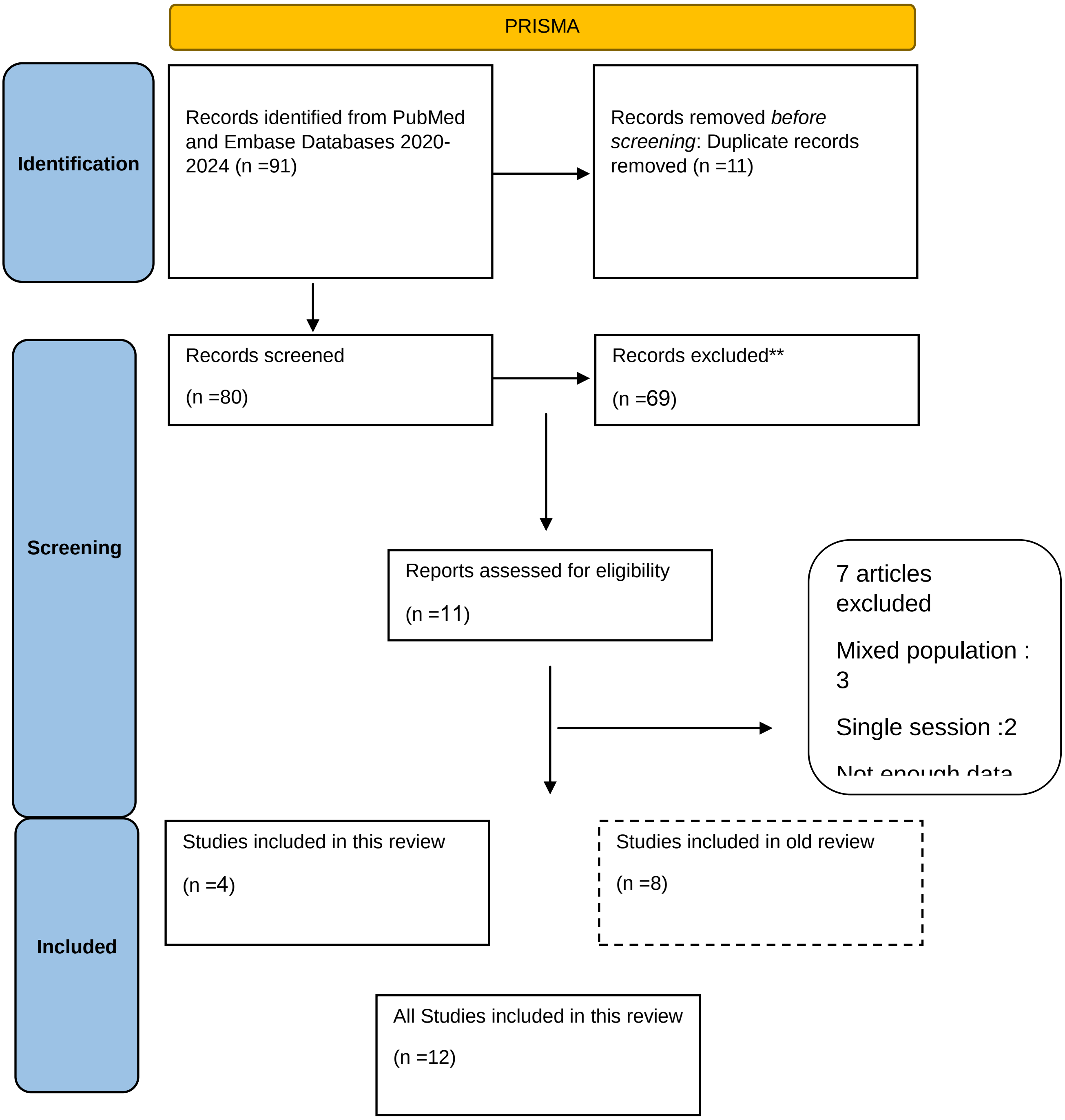


PRISMA Flow diagram showing studies related to the Oscillatory Positive Expiratory Pressure (OPEP) devices in COPD. *From:* Page MJ, McKenzie JE, Bossuyt PM, Boutron I, Hoffmann TC, Mulrow CD, et al. The PRISMA 2020 statement
