# Supplementary material for: Use of oscillatory positive expiratory pressure (OPEP) devices to augment sputum clearance in COPD: An updated systematic review and meta-analysis
Source: Chron Respir Dis. 2026 Jun 23;23:14799731261463730. doi: 10.1177/14799731261463730 (PMC13305770; doi:10.1177/14799731261463730)
Supplement: Supplemental material - Use of oscillatory positive expiratory pressure (OPEP) devices to augment sputum clearance in COPD: An updated systematic review and meta-analysis [file sj-pdf-3-crd-10.1177_14799731261463730.pdf]

Appendix 4 *Funnel plot for detection of publication bias* and risk of bias in all 12 studies

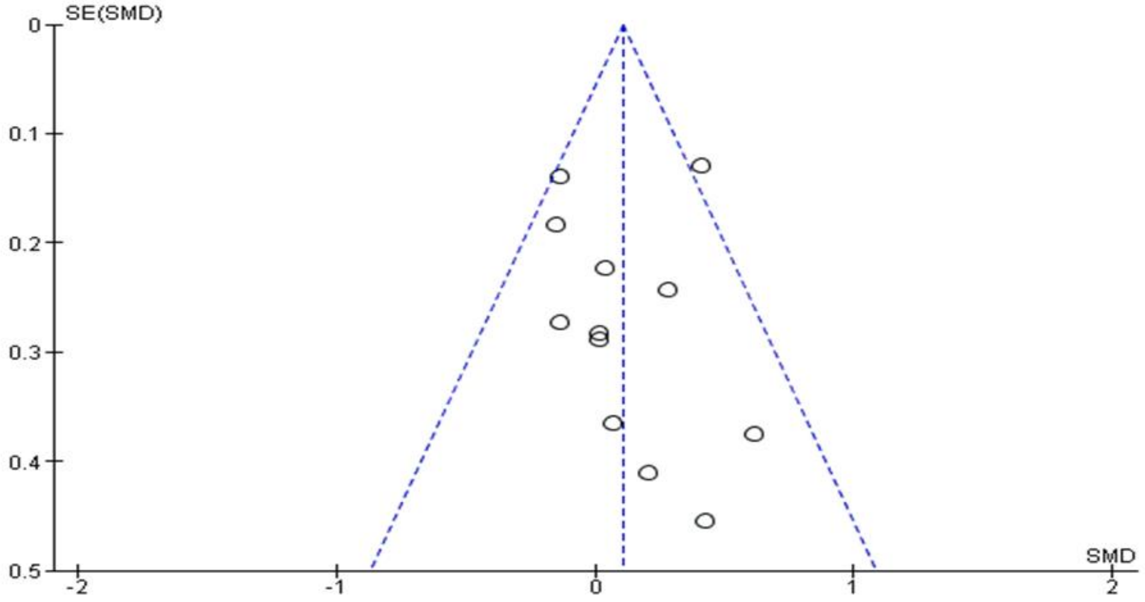


*Funnel plot for detection of publication bias. SMD, standardised mean difference*

| | Random sequence generation (selection bias) | Allocation concealment (selection bias) | Blinding of participants and personnel (performance bias) | Blinding of outcome assessment (detection bias) | Incomplete outcome data (attrition bias) | Selective reporting (reporting bias) | Other bias |
|---|---|---|---|---|---|---|---|
| Aggarwal et al | ? | ? | – | – | – | – | – |
| Alghamdi et al | + | + | + | + | + | + | ? |
| Cegla et al | ? | ? | – | – | + | – | – |
| Daynes et al | + | + | ? | + | + | + | + |
| McCarroll et al | + | + | ? | + | ? | + | ? |
| Nicolini et al | + | + | + | + | ? | + | ? |
| Sethi et al | + | + | – | – | + | + | – |
| Shamakh et al | + | + | – | + | + | + | + |
| Svenningsen et al | + | + | – | + | ? | + | ? |
| Weiner et al | + | ? | ? | + | ? | – | ? |
| Wolkove et al | ? | + | – | – | ? | + | ? |
| Xu et al | + | + | – | + | + | + | ? |
