# Supplementary material for: Use of oscillatory positive expiratory pressure (OPEP) devices to augment sputum clearance in COPD: An updated systematic review and meta-analysis
Source: Chron Respir Dis. 2026 Jun 23;23:14799731261463730. doi: 10.1177/14799731261463730 (PMC13305770; doi:10.1177/14799731261463730)
Supplement: Supplemental material - Use of oscillatory positive expiratory pressure (OPEP) devices to augment sputum clearance in COPD: An updated systematic review and meta-analysis [file sj-pdf-4-crd-10.1177_14799731261463730.pdf]

Appendix 3 GRAD assessment for outcomes

Outcome	No. of Studies (Design)	Effect Estimate	Certainty of Evidence (GRADE)	Reasons for Downgrading
**Health-Related Quality of Life (HRQoL)**	5 RCTs	SMD –0.56 (95% CI: –1.15 to 0.03)	Very Low	Risk of bias, high heterogeneity, imprecision
**Exacerbations**	4 RCTs	OR 0.39 (95% CI: 0.23 to 0.64)	Moderate	Some risk of bias, incomplete reporting
**Antibiotic Use**	1 RCT	OR 0.05 (95% CI: 0.01 to 0.38)	Very Low	Single study, indirectness, imprecision
**Exercise Capacity (6MWD)**	6 RCTs	MD +49.39 m (95% CI: 25.05 to 73.73 m)	Moderate	Moderate heterogeneity
**Lung Function (FVC%)**	6 RCTs	MD +3.06% (95% CI: 0.18 to 5.95%)	Moderate	Inconsistency between subgroups
**Sputum Clearance**	2 RCTs	Both favored OPEP (different scales)	Low	Few studies, different outcome measures
**Acceptance / Completion / Dropout**	4 RCTs	High acceptance; lower dropout in OPEP arms	Low	Incomplete reporting, varying definitions
