# Supplementary material for: Use of oscillatory positive expiratory pressure (OPEP) devices to augment sputum clearance in COPD: An updated systematic review and meta-analysis
Source: Chron Respir Dis. 2026 Jun 23;23:14799731261463730. doi: 10.1177/14799731261463730 (PMC13305770; doi:10.1177/14799731261463730)
Supplement: Supplemental material - Use of oscillatory positive expiratory pressure (OPEP) devices to augment sputum clearance in COPD: An updated systematic review and meta-analysis [file sj-pdf-6-crd-10.1177_14799731261463730.pdf]

Appendix 6. List of Abbreviations

**Abbreviation**	Full Term
**AECOPD**	Acute Exacerbation of Chronic Obstructive Pulmonary Disease
**ATS**	American Thoracic Society
**BMI**	Body Mass Index
**CI**	Confidence Interval
**COPD**	Chronic Obstructive Pulmonary Disease
**ERS**	European Respiratory Society
**FEV_1**	Forced Expiratory Volume in One Second
**FVC**	Forced Vital Capacity
**GOLD**	Global Initiative for Chronic Obstructive Lung Disease
**GRADE**	Grading of Recommendations Assessment, Development and Evaluation
**HRQoL**	Health-Related Quality of Life
**I^2**	I-squared statistic
**MD**	Mean Difference
**NICE**	National Institute for Health and Care Excellence
**OPEP**	Oscillatory Positive Expiratory Pressure
**OR**	Odds Ratio
**PEQ**	Patient Evaluation Questionnaire
**PEP**	Positive Expiratory Pressure
**PRISMA**	Preferred Reporting Items for Systematic Reviews and Meta-Analyses
**PROSPERO**	International Prospective Register of Systematic Reviews
**RCT**	Randomized Controlled Trial
**SD**	Standard Deviation
**SMD**	Standardized Mean Difference
**6MWD**	Six-Minute Walk Distance
